# Polydatin Protecting Kidneys against Hemorrhagic Shock-Induced Mitochondrial Dysfunction* via* SIRT1 Activation and p53 Deacetylation

**DOI:** 10.1155/2016/1737185

**Published:** 2016-02-24

**Authors:** Zhenhua Zeng, Zhongqing Chen, Siqi Xu, Qin Zhang, Xingmin Wang, Youguang Gao, Ke-seng Zhao

**Affiliations:** ^1^Department of Critical Care Medicine, Nanfang Hospital, Southern Medical University, Guangzhou 510515, China; ^2^Guangdong Key Lab of Shock and Microcirculation Research, Department of Pathophysiology, Southern Medical University, Guangzhou 510515, China; ^3^Department of Anesthesiology, The First Affiliated Hospital of Fujian Medical University, Fuzhou, Fujian 350005, China

## Abstract

*Objectives.* To ascertain if mitochondrial dysfunction (MD) of kidney cells is present in severe hemorrhagic shock and to investigate whether polydatin (PD) can attenuate MD and its protective mechanisms.* Research Design and Methods. *Renal tubular epithelial cells (RTECs) from rat kidneys experiencing HS and a cell line (HK-2) under hypoxia/reoxygenation (H/R) treatment were used. Morphology and function of mitochondria in isolated RTECs or cultured HK-2 cells were evaluated, accompanied by mitochondrial apoptosis pathway-related proteins.* Result. *Severe MD was found in rat kidneys, especially in RTECs, as evidenced by swollen mitochondria and poorly defined cristae, decreased mitochondrial membrane potential (ΔΨ*m*), and reduced ATP content. PD treatment attenuated MD partially and inhibited expression of proapoptotic proteins. PD treatment increased SIRT1 activity and decreased acetylated-p53 levels. Beneficial effect of PD was abolished partially when the SIRT1 inhibitor Ex527 was added. Similar phenomena were shown in the H/R cell model; when pifithrin-*α* (p53 inhibitor) was added to the PD/Ex527 group, considerable therapeutic effects were regained compared with the PD group apart from increased SIRT1 activity.* Conclusions. *MD is present in severe HS, and PD can attenuate MD of RTECs* via* the SIRT1-p53 pathway. PD might be a promising therapeutic drug for acute renal injury.

## 1. Introduction

During hemorrhagic shock and reperfusion (HS/R), reduction of systemic perfusion and local perfusion is a notable feature and can lead to multiple-organ failure, including acute kidney injury (AKI) [1]. Mitochondrial dysfunction (MD) is one of the main reasons for AKI. It has been demonstrated that ischemia and hypoxia, dysfunction in the microcirculation, release of reactive oxygen species, and endogenous apoptotic pathways are associated with mitochondrial disorders [2]. Apoptosis is an active energy-consuming process and is regulated strictly. p53 is a tumor suppressor that plays an important part in apoptosis regulation. The quantity, stability, and activity of p53 are regulated by various posttranslational modifications, including phosphorylation, ubiquitination, and acetylation.

The sirtuin family is involved in transcriptional repression, chromatin silencing, and the pathogenesis of renal diseases [3]. Silent information regulator (SIRT)1 is a nicotinamide adenine dinucleotide- (NAD^+^-) dependent histone deacetylase, and the p53 gene was the first SIRT1 deacetylation nonhistone target to be discovered [4, 5]. Numerous studies have demonstrated the deacetylation role of SIRT1 on p53 and downregulation of p53 activity [4, 6].

In recent years, resveratrol has been reported widely to be an activator of SIRT1 [7] and has been shown to reduce ischemia-reperfusion injury in kidneys [8]. In addition, resveratrol has been shown to deacetylate p53 by activating the SIRT1 pathway, reducing cisplatin-induced injury to proximal tubular epithelial cells in mice [9], and doxorubicin-induced myocardial apoptosis [10]. Therefore, sirtuin-family members and their activators may be promising therapeutic targets for ischemia-reperfusion injury.

Polydatin (PD; also known as “piceid”) is an active ingredient extracted from the roots of the traditional Chinese herb* Polygonum cuspidatum*. The chemical structure of PD has been identified to be 3,4,5-trihydroxystilbene-3-*β*-D-glucoside (Supplementary Figure 1 in Supplementary Material available online at http://dx.doi.org/10.1155/2016/1737185). Several studies have shown that PD possesses important therapeutic effects in animal models of shock. It has been reported that PD can attenuate damage against ischemia-reperfusion injury in multiple organs [11–13]. Our research team revealed that PD can alleviate oxidative stress and can protect mitochondria in vascular smooth muscle cells [14], neurons [15], and hepatocytes [16] against severe shock. As a* trans*-resveratrol analog, PD can reduce generation of oxygen free radicals in mitochondrial electron transport respiratory chain complex III, thereby demonstrating a direct role in mitochondrial protection [15]. Recently, our research team demonstrated that PD can alleviate injury to the small intestine through SIRT1 activation [17]. Whether PD can attenuate renal injury in hemorrhagic shock (HS) has not been reported, and its exact mechanism of action is not known.

We wished to evaluate mitochondrial function in kidney cells and to investigate the potential effects of PD on mitochondrial protection and the SIRT1-p53 pathway. Thus, an animal model of HS was created and hypoxia/reoxygenation (H/R) human kidney- (HK-) 2 cells used. We observed severe MD in renal tubular epithelial cells (RTECs) after HS/R that was related to the SIRT1-p53 pathway. Moreover, PD may be a new activator that results in a reduction of acetylated-p53 (acetyl-p53) levels, leading to attenuation of mitochondrial damage.

## 2. Materials and Methods

### 2.1. Reagents and Antibodies

PD and its specific vehicle (ethanol 70%, propylene glycol 20%, and NaHCO_3_ 10%) were supplied by Neptunus Co. (Shenzhen, Guangdong, China); its purity was >99.5%. MitoProbe*™* JC-1(5,5′,6,6′-Tetrachloro-1,1′,3,3′-tetraethyl-imidacarbocyanine iodide), calcein-AM, and MitoTracker*™* (Thermo Fisher, Carlsbad, CA) were purchased from Molecular Probes (Invitrogen, CA). The CellTiter-Glo assay and a terminal deoxynucleotidyl transferase dUTP nick-end labeling (TUNEL) staining kit were supplied from Promega Corp. (Madison, WI). A mitochondrial/cytosolic protein extraction kit was purchased from BestBio Co. (Beijing, China). Antibodies against cytokeratin 18, p53 upregulated modulator of apoptosis- (PUMA-) *α*, B-cell lymphoma- (Bcl-) 2, Bcl-2-associated X protein (Bax), and a SIRT1 activity assay kit were obtained from Abcam (Cambridge, UK). Antibodies against cleaved caspase-3 were purchased from Cell Signaling Technology Inc. (Danvers, MA). Antibodies against SIRT1 and p53 were supplied by Santa Cruz Biotechnology (Santa Cruz, CA). An immunoprecipitation kit was purchased from Proteintech (Chicago, IL). Antibodies against acetyl-p53 and cytochrome C, as well as a fluorescein isothiocyanate (FITC) annexin V apoptosis kit, were obtained from BD Biosciences (San Jose, CA). The human renal proximal tubular epithelial cell line HK-2 was supplied by American Type Culture Collection (Manassas, VA). Cell Count Kit-8 (CCK-8) was purchased from Dojindo Co. (Shanghai, China). Secondary polyclonal rabbit anti-rat immunoglobulin/FITC and an immunoprecipitation kit were obtained from Proteintech Co. Polyvinylidene fluoride (PVDF) membranes were purchased from Millipore (Billerica, MA). All other chemicals were from Sigma-Aldrich (Saint Louis, MO).

### 2.2. Establishment of a HS/R Model in Rats

The present study was undertaken in strict accordance with recommendations from the* Guide for the Care and Use of Laboratory Animals* (National Institutes of Health, Bethesda, MD, USA). The study protocol was approved by the Ethics Committee for Animal Experiments of the University of Southern Medical University (Guangzhou, China).

Adult specific pathogen-free Sprague-Dawley rats (male or female; 180–220 g; 7-8 weeks) were obtained from the Laboratory Animal Centre of Southern Medical University. They were housed in metabolic cages under controlled conditions (25°C; 12-h light-dark cycle). Animals had free access to standard rat chow and tap water. All efforts were made to minimize animal suffering and to reduce the number of rats used.

Thirty-two rats were anesthetized with a mixture of 13.3% urethane and 0.5% chloralose-*α* (0.65 mL/100 g body weight). Rats were subjected to HS for 120 min followed by resuscitation with shed blood as undertaken by our research team previously, with slight modifications [15]. Briefly, after implantation of PE-50 catheters in arterial and venous passages, the mean arterial pressure (MAP) was recorded using measurement equipment from PowerLAB (AD Instruments, Sydney, Australia). Rats were bled through a syringe to obtain a MAP of 30 mmHg within 10 min, which was maintained for the next 2 h by withdrawal or reinfusion of stored blood. PD, vehicle, or PD/Ex527 (Ex527 is an inhibitor of SIRT1) was administered* via* the intravenous route within 10 min and, 10 min later, shed blood was reinfused. Rats were divided randomly into four groups: (i) control (sham; rats were anesthetized and underwent surgery without any other treatments); (ii) vehicle (rats were subjected to HS to maintain the MAP at 30 mmHg for 120 min, followed by administration of vehicle (0.3 mL) and infusion of shed blood); (iii) PD (rats were subjected to HS for 120 min, followed by administration of PD (30 mg/kg) dissolved in 0.3 mL solvent and infusion of shed blood (PD dose administered was based on our previous studies [14, 15])); (iv) PD/Ex527 (rats were subjected to HS for 120 min, followed by administration of PD (30 mg/kg) and Ex527 (5 mg/kg) [18] dissolved in 0.3 mL vehicle and infusion of shed blood). Two hours after reinfusion of shed blood, 1 mL of blood from each rat was collected from the femoral artery and centrifuged for measurement of renal function. Levels of blood urea nitrogen (BUN) and creatinine (Cr) were measured by an automatic biochemical analyzer (AU5400; Olympus, Tokyo, Japan). Then, all rats (eight in each group) were killed by cervical dislocation.

### 2.3. Morphological Observation and Immunohistochemistry of Kidney Tissue

One portion of kidney tissue was used for morphological observation, immunohistochemistry, and protein extraction from renal cortices. Kidney samples were prepared and observed using transmission electron microscopy (TEM) using methods described previously [15]. Kidney tissue was fixed in neutral-buffered formalin, embedded in paraffin, and cut into transverse sections (thickness, 4 mm) for immunohistochemical studies. Expression of Bcl-2, Bax, and SIRT1 in tissues was visualized using an immunohistochemical method (EnVision*™*; Dako, Copenhagen, Denmark) with rabbit polyclonal anti-rat antibodies. Working concentrations of antibodies against Bcl-2, Bax, and SIRT1 were 1 : 250, 1 : 250, and 1 : 100, respectively.

### 2.4. Isolation of RTECs and Detection of Mitochondrial Function

The other portion of kidney tissue was used for isolation of RTECs by a method described previously, with slight modifications [19, 20]. Briefly, the cortex was cut into fragments. Cells were dissociated by incubation for 30 min at 37°C with 1 mg/mL type-I collagenase. Red blood cells were removed by lysis. RTECs were separated by Percoll gradient density centrifugation [21]. Purity of RTECs was examined by immunostaining with cytokeratin-18 and Hoechst dye [22] (Supplementary Figure 2). Isolated cells were used for detection of mitochondrial function (mitochondrial membrane potential (ΔΨ*m*), cellular level of adenosine triphosphate (ATP), mitochondrial permeability transition pore (mPTP), and lysosomal stability), as described previously [15].

### 2.5. Cell Culture and H/R Treatment

The human renal proximal tubular epithelial line HK-2 was cultured in Dulbecco's modified Eagle's medium (DMEM) supplemented with 10% (*v*/*v*) heat-inactivated fetal bovine serum (FBS) and 1.0 mmol/L sodium pyruvate at 37°C in a humidified atmosphere containing 5% CO_2_.

Before experimentation, cultured cells at ≈80% confluency were serum-starved for 24 h in DMEM/F12 supplemented with 0.1% FBS. Various concentrations of PD were added to cells 2 h before H/R. Then, cells were exposed to different durations of H/R and cell viability determined using a CCK-8 kit in accordance with manufacturer instructions. PD (50 *μ*M) was selected as the “ideal” concentration. Forty-eight hours of hypoxia (5% CO_2_, 1% O_2_, and 94% N_2_) followed by 2 h of reoxygenation (5% CO_2_, 21% O_2_, and 74% N_2_) was chosen for further study (Supplementary Figure 3).

Cells were divided randomly into five groups: (i) control (cells were incubated in normoxic conditions (5% CO_2_, 21% O_2_, and 74% N_2_) without treatment with PD or H/R); (ii) vehicle (cells pretreated with a specific vehicle of PD were exposed to H/R); (iii) PD (cells pretreated with PD were exposed to H/R); (iv) PD/Ex527 (cells pretreated with PD and Ex527 (selective inhibitor of SIRT1 and used at 10 *μ*M [23]) were exposed to H/R); (v) PD/Ex527/pifithrin- (PFT-) *α* (cells pretreated with PD, Ex527, and PFT-*α* (p53 inhibitor and used at 10 *μ*M [24]) were exposed to H/R).

### 2.6. Immunofluorescence Studies

After drug administration and creation of a H/R model, HK-2 cells were incubated with staining solution containing a MitoTracker probe for 30 min. After staining, cultured cells were fixed with ice-cold 4% paraformaldehyde solution for 20 min and permeabilized in 0.2% TritonX-100 for 20 min. Cells were blocked with 1% bovine serum albumin for 30 min at room temperature. Primary rabbit polyclonal anti-SIRT1 and p53 (1 : 200 dilution) were incubated with cells for >16 h at 4°C, and secondary polyclonal rabbit anti-rat immunoglobulin/FITC was added to cells for 1 h at 37°C. Finally, 4′,6-diamidino-2-phenylindole was added for nuclear staining. Cells were observed and analyzed using a confocal laser scanning microscope (LSM 780 NLO; Carl Zeiss, Jena, Germany).

### 2.7. Flow Cytometry

Indices related to mitochondrial function (ΔΨ*m*, mPTP, and lysosomal stability) were analyzed using a flow cytometer (FACSVerse; BD Biosciences, San Jose, CA) using methods described previously [15] accompanied by determination of acetyl-p53 levels.

### 2.8. Western Blotting

Samples of renal cortex tissue and HK-2 cells were lysed in radioimmunoprecipitation assay buffer. Total protein and cytoplasmic protein were extracted after centrifugation and mixed with 5x sodium dodecyl sulfate (SDS) sample buffer. Samples were separated by SDS-polyacrylamide gel electrophoresis using 8–12% acrylamide gels and transferred to PVDF membranes. After incubation with primary antibodies (against PUMA-*α*, Bax, Bcl-2, cytochrome C, cleaved caspase-3, and SIRT1) and secondary antibodies, protein bands were detected using chemiluminescence detection reagents. acetyl-p53 levels were measured on immunoprecipitated p53 protein.

### 2.9. SIRT1 Activity

Fresh lysed renal cortex tissues or cells were immunoprecipitated with antibody against SIRT1 (Santa Cruz Biotechnology) and normalized SIRT1 protein content in each group. Then, a reaction mixture containing fluorosubstrate peptide solution and protein-A agarose beads was added. NAD-dependent deacetylase activity was measured based on fluorescence intensity at 1-2 min intervals at an excitation wavelength of 350 and emission wavelength of 460 nm on a SpectraMax M5 system (Thermo Fisher). Activity was presented as a relative value compared with that of the control group.

### 2.10. Statistical Analyses

Results are the mean ± standard deviation and were analyzed using SPSS v20.0 (IBM, Armonk, NY, USA). The homogeneity of variance (Levene's) test was used to ascertain if groups had equal variance. If Levene's test indicated homogeneity of variance (*P* > 0.1), the unpaired* t*-test was used to compare values between two groups. One-way ANOVA was done to compare differences in multiple groups after Tukey's honestly significant difference multiple-comparison test and *P* < 0.05 was considered significant. When equal variances were not assumed (based on Levene's test; *P* < 0.1), Dunnett's T3* post hoc* comparisons were used for robust tests of equality of mean values. *P* < 0.05 was considered significant.

## 3. Results

### 3.1. PD Activates SIRT1 Activity and Deacetylates p53

Based on a review of the effect of PD in our previous work [17], it was hypothesized that PD could activate SIRT1 activity, deacetylate p53, and inhibit apoptosis and serve as a therapeutic agent against HS. Ex527 (selective inhibitor of SIRT1) and PFT-*α* (reversible inhibitor of p53) were introduced to test this hypothesis.

First, the effect of PD on normal rats was observed. After 7-day intraperitoneal administration of PD, Ex527 (5 mg/kg), or solvent, protein expression and activity of SIRT1 were elevated in the PD group but reduced in the Ex527 group (Supplementary Figures 4A, 4B). Moreover, a PD/Ex527/PFT-*α* group was additionally added to HK-2 cells based on animal experimental grouping to evaluate the possible relationship between SIRT1 and p53. After preadministration of vehicle, PD, Ex527 (10 *μ*M), or PFT-*α* (10 *μ*M) for 7 days, protein expression and activity of SIRT1 were elevated in the PD group but reduced in the Ex527 group. PFT-*α* did not appear to activate SIRT1 (Supplementary Figures 4C, 4D).

**Figure 1:**
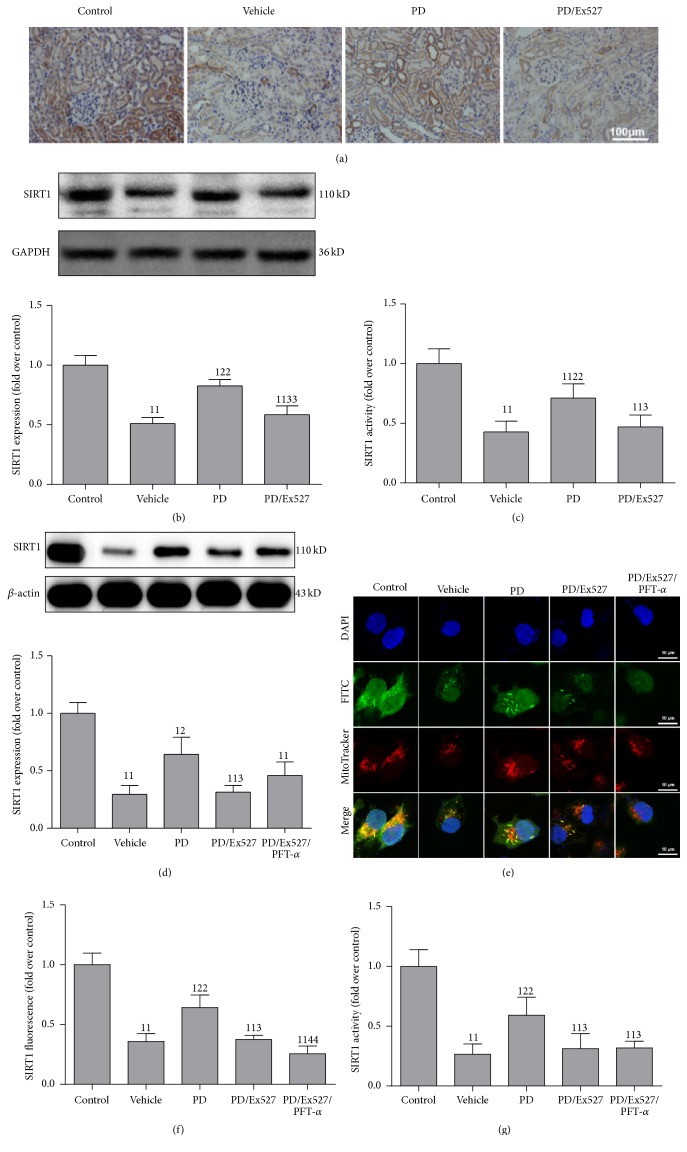
Effect of polydatin (PD) administration on the protein expression and activity of SIRT1 in the renal cortex after HS/R and in HK-2 cells after H/R, respectively. (a) Immunohistochemistry of SIRT1; (b) expression of SIRT1 protein (*n* = 3); (c) SIRT1 activity in the kidney cortex (*n* = 6); (d) expression of SIRT1 protein determined using western blotting (*n* = 3); (e-f) SIRT1 immunocytochemistry using confocal microscopy (original magnification, ×630); (g) determination of SIRT1 activity (*n* = 6). ^11^ *P* < 0.01, ^1^ *P* < 0.05 compared with the value of the vehicle group; ^22^ *P* < 0.01, ^2^ *P* < 0.05 compared with the value of the vehicle group; ^33^ *P* < 0.01, ^3^ *P* < 0.05 compared with the value of the PD group; ^44^ *P* < 0.01 compared with the value of the PD/Ex527 group. GAPDH, glyceraldehyde 3-phosphate dehydrogenase; HS/R, hemorrhagic shock and reperfusion; H/R, hypoxia/reoxygenation; SIRT1, silent information regulator 1; HK-2, human kidney-2.

Subsequently, the effect of PD on SIRT1 in the rat model of HS/R or the cell model of H/R was elevated. As expected, protein expression (Figures 1(a) and 1(b)) and activity (Figure 1(c)) of SIRT1 in the renal cortex decreased after HS/R in the vehicle group compared with that in the control group. PD treatment partially restored the protein expression and activity of SIRT1 compared with that in the vehicle group. However, the beneficial effect of PD on SIRT1 was almost abolished after Ex527 addition (Figures 1(a)–1(c)). In agreement with the animal study, protein expression and activity of SIRT1 (which were decreased in the vehicle group after H/R) were restored by PD administration in HK-2 cells. However, when Ex527 was added to the PD/Ex527 group, the beneficial effect of PD on SIRT1 was blocked partially, suggesting that the effect of PD was related to SIRT1 activation. Moreover, the protein expression and activity of SIRT1 were also decreased in the PD/Ex527/PFT-*α* group compared with the PD group, suggesting that PFT-*α* does not affect protein expression and activity of SIRT1 (Figures 1(d)–1(g)).

**Figure 2:**
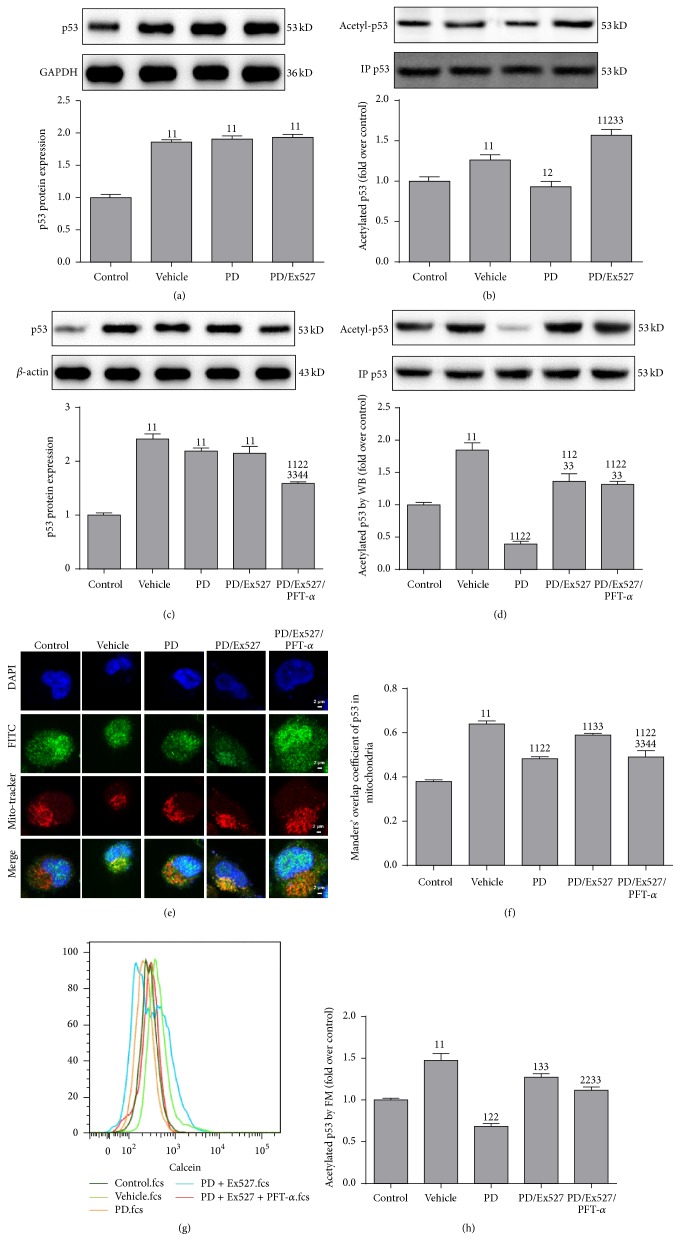
Effect of polydatin (PD) administration on the protein expression and acetylation of p53 in the renal cortex after HS/R and in HK-2 after H/R, respectively. (a) Expression of p53 protein and (b) determination of acetyl-p53 levels on purified p53 (*n* = 5) in the renal cortex; (c) expression of p53 protein determined using western blotting (*n* = 5); (d) expression of acetyl-p53 determined in purified p53 using western blotting (*n* = 3); (e, f) intracellular redistribution of p53 protein determined using confocal microscopy (original magnification, ×630); (g, h) expression of acetyl-p53 determined using flow cytometry. ^11^ *P* < 0.01, ^1^ *P* < 0.05 compared with the value of the vehicle group; ^22^ *P* < 0.01, ^2^ *P* < 0.05 compared with the value of the vehicle group; ^33^ *P* < 0.01 compared with the value of the PD group; ^44^ *P* < 0.01 compared with the value of the PD/Ex527 group. GAPDH, glyceraldehyde 3-phosphate dehydrogenase; H/R, hypoxia/reoxygenation; IP, immunoprecipitation.

In addition to SIRT1, the most reported downstream target, p53, was also determined. Expressions of p53 protein (Figure 2(a)) and acetyl-p53 were increased in the shocked group (Figure 2(b)). Treatment with PD reduced the acetyl-p53 level (Figures 2(a) and 2(b)). However, the acetyl-p53 level was reelevated after Ex527 addition in the PD/Ex527 group. Further studies were undertaken in HK-2 cells. Mitochondrial localization and protein expression of p53 were observed using immunocytochemistry (Figures 2(e) and 2(f)) as well as western blotting (Figures 2(c) and 2(d)) and flow cytometry (Figures 2(g) and 2(h)), respectively. In HK-2 cells experiencing H/R, increased translocation of p53 from the cytosol to mitochondria (mitochondrial transfer) was observed in addition to increased expression of p53 protein and acetyl-p53. SIRT1 activation in the PD group resulted in less p53 being located in mitochondria and less expression of acetyl-p53 in HK-2 cells. However, this phenomenon was almost reversed when Ex527 was added to the PD/Ex527 group, which was similar to that observed in the vehicle group. Importantly, localization of p53 in mitochondria and acetyl-p53 levels were reduced again when PFT-*α* was added to the PD/Ex527/PFT-*α* group. These results suggested that PD increased SIRT1 activity and reduced acetyl-p53 levels and mitochondrial transfer of p53.

### 3.2. PD Suppresses Apoptosis

**Figure 3:**
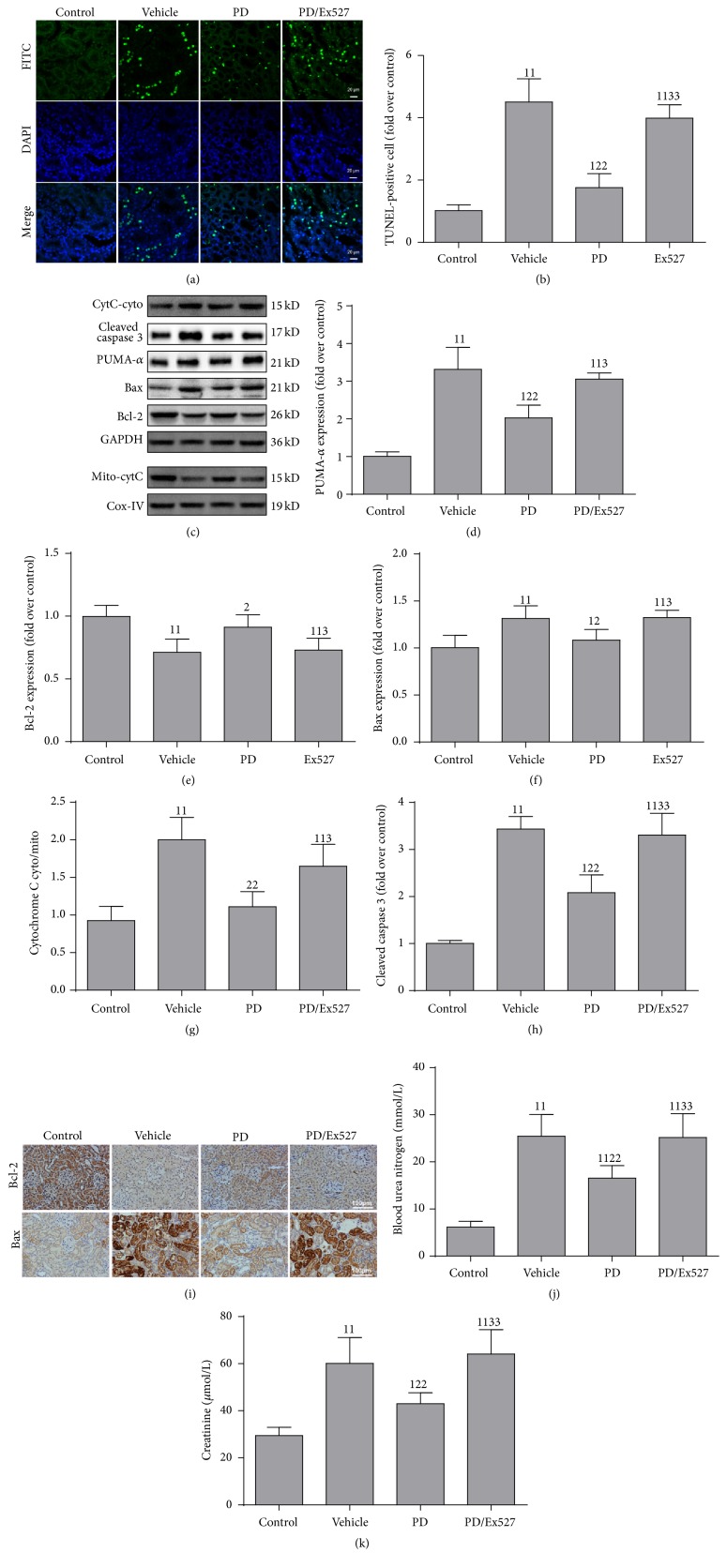
Effect of polydatin (PD) administration on mitochondrial apoptosis pathway-related protein expression and renal function after hemorrhagic shock. (a, b) TUNEL staining of renal cortex (original magnification, ×400); (c) western blotting of expression of PUMA-*α*, Bcl-2, Bax, cytoplasm cytochrome C (cyto-cytC), and mitochondrial cytochrome C (mito-cytC), and cleaved caspase-3; (d) quantitation of PUMA-*α*, (e) Bax, (f) Bcl-2, and (h) cleaved caspase-3 protein, *n* = 3; (g) cytoplasm/mitochondria (cyto/mito) ratio of cytochrome C (*n* = 5 in each group); (i) immunohistochemistry of the proapoptotic protein Bax and antiapoptotic protein Bcl-2; (j) determination of levels of blood urea nitrogen and (k) creatinine, *n* = 6. ^11^ *P* < 0.01, ^1^ *P* < 0.05 compared with the value of the vehicle group; ^22^ *P* < 0.01, ^2^ *P* < 0.05 compared with the value of the vehicle group; ^33^ *P* < 0.01, ^3^ *P* < 0.05 compared with the value of the PD group. DAPI, 4′,6-diamidino-2-phenylindole; GAPDH, glyceraldehyde 3-phosphate dehydrogenase; FITC, fluorescein isothiocyanate; TUNEL, terminal deoxynucleotidyl transferase dUTP nick-end labeling.

Due to effect of PD on p53 deacetylation (molecule-orchestrated apoptosis), we tested apoptosis in renal tissue and HK-2 cells. The number of TUNEL-positive cells in the renal cortex increased significantly in shocked rats (vehicle group, Figures 3(a) and 3(b)). Also expression of proapoptotic proteins (Bax, PUMA-*α*) and the cytoplasm/mitochondria (cyto/mito) ratio of cytochrome C and cleaved caspase-3 was elevated, and expression of the antiapoptosis protein Bcl-2 was diminished (Figures 3(c)–3(i)). PD treatment increased levels of Bcl-2 protein and reduced expression of the proapoptotic proteins PUMA-*α* and Bax and the cyto/mito ratio of cytochrome C and cleaved caspase-3, as well as apoptosis. However, in the PD/Ex527 group, the number of apoptotic cells was increased. These results implied that PD inhibited apoptosis (Figures 3(a)–3(i)). Consistent with determination of apoptosis, indices of renal function (BUN, Cr) that were elevated remarkably in shocked rats were reduced by PD administration (Figures 3(j) and 3(k)).

**Figure 4:**
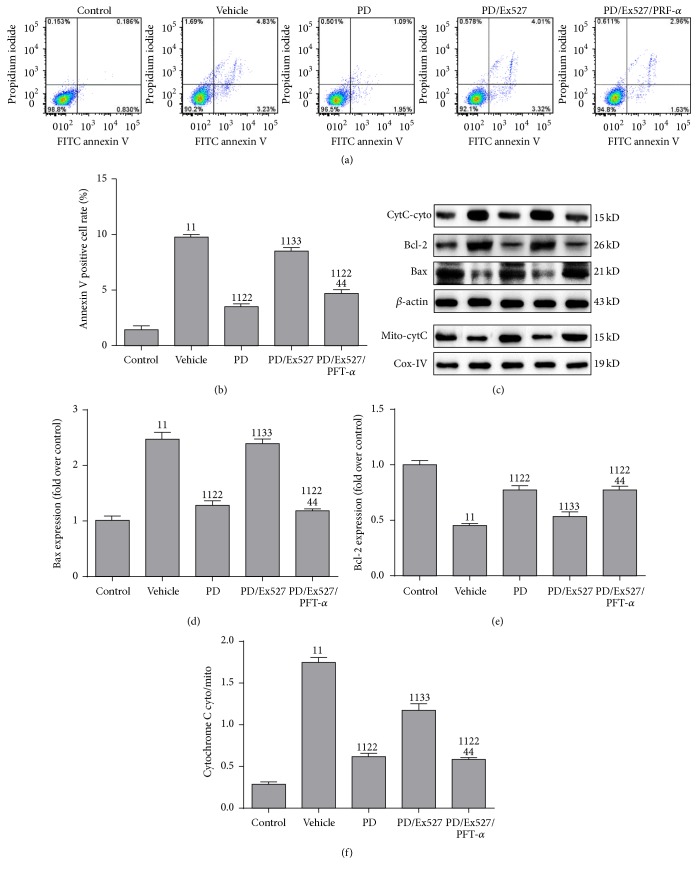
Effect of polydatin (PD) administration on mitochondrial apoptosis pathway-related protein expression after hypoxia and reoxygenation. (a-b) Apoptosis measured using an annexin V and propidium iodide kit (apoptosis was reflected by the percentage of annexin V positive cells); (c) western blotting of expression of Bax, Bcl-2, cytoplasmic cytochrome C (cyto-cytC), and mitochondrial cytochrome C (mito-cytC) in HK-2 cells; (d–f) quantification of the band intensity of Bax, Bcl-2, and cyto/mito ratio of cytochrome C. The result is a representative band of three independent experiments. ^11^ *P* < 0.01 compared with the value of the vehicle group; ^22^ *P* < 0.01 compared with the value of the vehicle group; ^33^ *P* < 0.01 compared with the value of the PD group; ^44^ *P* < 0.01 compared with the value of the PD/Ex527 group. HK-2, human kidney-2.

In agreement with the animal-model study, a similar result was found in the HK-2 cell model, as evidenced by the increased number of apoptosis-positive cells, elevated level of Bax protein, and increased cyto/mito ratio of cytochrome C (Figure 4). As expected, PD administration reduced apoptosis, decreased expression of Bax, restored expression of Bcl-2, and diminished the cyto/mito ratio of cytochrome C. However, upon Ex527 addition, apoptosis was increased again, as evidenced by the increased number of apoptotic cells, increased expression of Bax and the cyto/mito ratio of cytochrome C, and decreased expression of Bcl-2. As expected, when PRF-*α* was added to the PD/Ex527/PFT-*α* group, apoptosis was reduced markedly, accompanied by reduced expression of Bax and the cyto/mito ratio of cytochrome C and increased expression of Bcl-2.

### 3.3. PD Ameliorates MD

**Figure 5:**
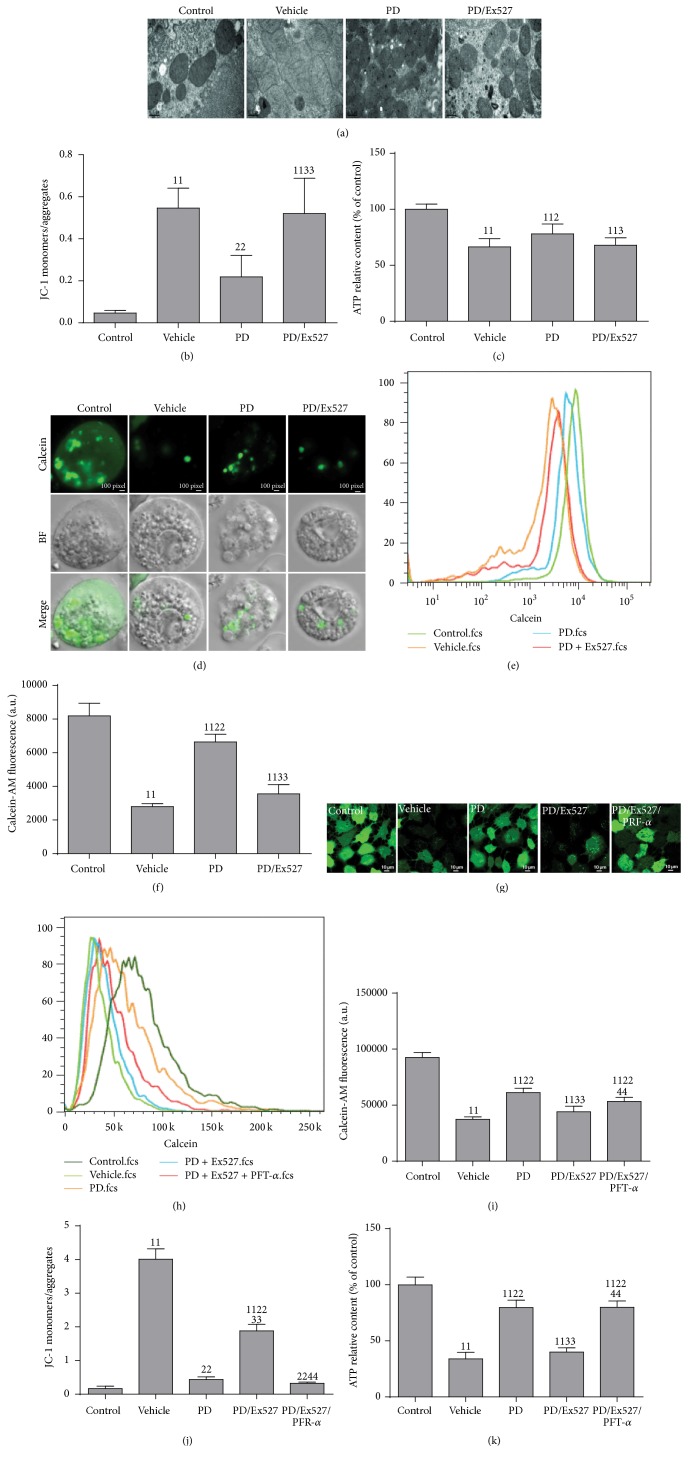
Effect of polydatin (PD) administration on the morphology and function of mitochondria in RTECs after HS/R and in HK-2 cells after H/R, respectively. (a) Ultrastructural alterations in RTEC mitochondria were detected using TEM (original magnification, ×11,500); (b) quantification of RTEC mitochondrial depolarization expressed as changes in the JC-1 fluorescence monomer/aggregate ratio (*n* = 4–6); (c) intracellular level of ATP in RTECs (*n* = 8). (d–f) mPTP in RTECs was observed using confocal microscopy (original magnification, ×630) and quantified using flow cytometry (*n* = 5); (g–i) mPTP (reflected by the fluorescence intensity of calcein-AM) in HK-2 cells was observed using a microscope (original magnification, ×630) and quantified using flow cytometry; (j) quantification of depolarization of mitochondria in HK-2 cells as JC-1 monomer/aggregate using flow cytometry; (k) intracellular level of ATP in HK-2 cells using a luciferase-based assay (*n* = 3 in each group). ^11^ *P* < 0.01, ^1^ *P* < 0.05 compared with the value of the vehicle group; ^22^ *P* < 0.01, ^2^ *P* < 0.05 compared with the value of the vehicle group; ^33^ *P* < 0.01, ^3^ *P* < 0.05 compared with the value of the PD group; ^44^ *P* < 0.01 compared with the value of the PD/Ex527 group. HK-2, human kidney-2; H/R, hypoxia/reoxygenation; HS/R, hemorrhagic shock and reperfusion; mPTP, mitochondrial permeability transition pore; RTECs, renal tubular epithelial cells; TEM, transmission electron microscopy.

Finally, we measured the effect of PD on mitochondrial protection. Two hours after reinfusion of shed blood, rats were killed and kidneys were removed for observation of mitochondrial morphology using TEM. In the control group, normal mitochondria with preserved membranes and cristae were found. In contrast, mitochondrial damage was noted in some renal cells after HS/R, especially in RTECs, as evidenced by swollen and irregular-shaped mitochondria with disrupted and poorly defined cristae (Figure 5(a)). Thus, RTECs were isolated for determination of mitochondrial function (mPTP, ΔΨ*m*, and ATP levels). The calcein-AM/CoCl_2_ method was used to determine changes in mPTP. Weak fluorescence in mitochondria was detected in the shocked group using confocal microscopy, which was quantified further using flow cytometry, and suggested an opened mPTP (Figures 5(d)–5(f)). In addition, the ΔΨ*m* level was decreased as evidenced by an increased JC-1 monomer/aggregate ratio (Figure 5(b)). Moreover, the ATP level decreased (Figure 5(c)). Collectively, these results suggested severely damaged mitochondria after HS/R. Swollen mitochondria and irregular-shaped RTECs were restored partially by PD treatment under TEM. In addition, PD elevated the fluorescence intensity of calcein-AM in mitochondria, inhibited the loss of ΔΨ*m*, and restored ATP levels. However, all the beneficial effects of PD were blocked partially by Ex527 (Figure 5(a)–5(f)). These results suggested that PD could attenuate the mitochondrial injury to RTECs in HS/R rats.

Interestingly, in HK-2 cells experiencing H/R, severe mitochondrial damage was found in the vehicle group and was attenuated by PD administration, resulting in ameliorated mPTP (Figures 5(g)–5(i)), elevated ΔΨ*m* (Figure 5(j)), and increased ATP levels (Figure 5(k)). In contrast, mitochondrial damage was detected in the PD/Ex527 group. In addition, when PRF-*α* was added to the PD/Ex527/PFT-*α* group, mitochondrial function was restored partially as in the PD group (Figures 5(g)–5(k)).

## 4. Discussion

The present study elicited three main findings. First, mitochondrial damage in RTECs after severe HS/R, which is related to the decreased protein expression and activity of SIRT1, was demonstrated. Second, decreased SIRT1 activity may result in weaker deacetylation of p53, causing increased translocation of p53 from the cytoplasm to the mitochondria with release of mitochondrial proapoptotic proteins, which leads to mitochondrial damage and apoptosis. Third, PD is a new activator of SIRT1 that deacetylates p53, inhibits opening of the mPTP, suppresses a mitochondria-mediated apoptotic pathway, and attenuates renal injury against HS/R. This is the first study to demonstrate the critical role of SIRT1-p53 signaling in the mitochondrial-protective effect of PD.

Based on the severe MD of RTECs after HS/R, a possible molecular mechanism of MD was explored. Expression of mitochondrial proapoptotic proteins such as cytoplasmic cytochrome C and Bax was increased. It has been reported that p53 is an important transcription factor involved in regulation of mitochondrial apoptosis and that it can regulate the permeability of the outer membranes of mitochondria [19]. Therefore, the protein expression, intracellular distribution, and acetylation level of p53 in the renal cortex after HS/R were measured. In agreement with the results of AKI after ischemia-reperfusion [25, 26], it was found that the protein expression, mitochondrial translocation, and acetylation levels of p53 were increased and that the protein expression and activity of SIRT1 in the kidney cortex were decreased. When SIRT1 protein was activated by PD, SIRT1 activity was increased and acetyl-p53 level was decreased. These results suggest that the reduction in SIRT1 activity and downregulation of the p53 signaling pathway may be involved in mitochondrial damage after HS/R in RTECs.

**Figure 6:**
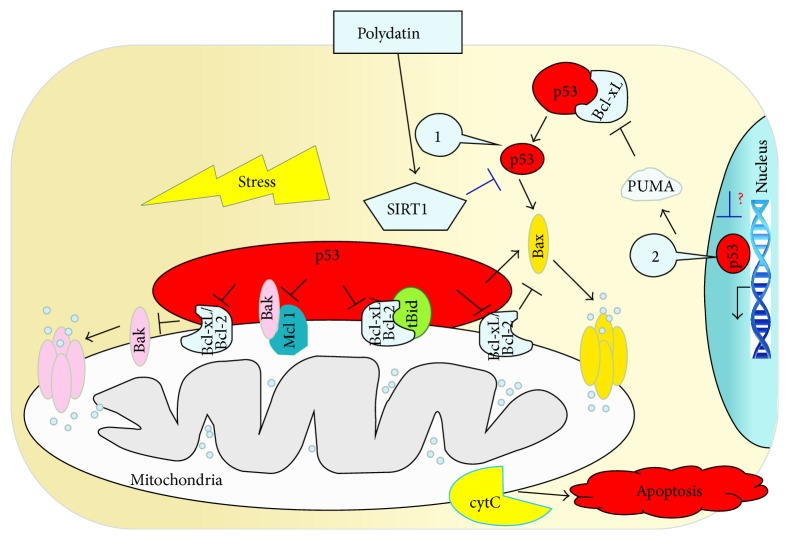
Effect of polydatin (PD) on the SIRT1-p53 signaling pathway. (1) p53 transcription nonindependent signaling pathway; (2) p53 transcription-independent signaling pathway. PD is likely to activate SIRT1, leading to inhibition of the p53 transcription nonindependent signaling pathway and mitochondrial-mediated apoptosis. SIRT1, silent information regulator 1.

SIRT1 is involved in regulation of two p53-mediated apoptotic pathways: transcription-dependent apoptosis and transcription-independent apoptosis [27]. p53 transcription-dependent apoptotic genes include Bax and PUMA. In contrast, p53 transcription-independent apoptosis is reliant upon direct interaction between p53 located in mitochondria and the antiapoptotic Bcl-2 family, which promotes release of cytochrome C from the inner membranes of mitochondria and finally activates apoptosis [28]. The present study demonstrated that PD elevated the protein expression and activity of SIRT1; reduced the protein expression, acetylation level, and mitochondrial translocation of p53; diminished expression of mitochondrial proapoptotic proteins; improved mitochondrial function. Based on* in vivo* and* in vitro* experiments, PD was used as an experimental drug. Once again, it was shown that the increased expression and activity of SIRT1 can reduce the acetylation level and mitochondrial translocation of p53, leading to a mitochondrial-protective effect. In one way, our study confirms that the SIRT1-p53 pathway is a new therapeutic target in shock-induced cell damage. In another way, we found that the SIRT1-p53 pathway is a molecular mechanism related to the mitochondrial-protective effect of PD (Figure 6).

The present study had limitations. First, only a single dose of PD as a therapeutic drug was studied in shocked rats. However, in a dose-response study in our previous report [15], a 30 mg/kg body-weight dose of PD showed better therapeutic effects than the low-dose group (15 mg/kg body weight), and it showed a nearly equal therapeutic effect to that in the high-dose group (45 mg/kg body weight). In addition, the effect of PD at a single time point (2 h after blood reinfusion) was observed because, based on our previous study, severe MD was evident at that time [14, 15]. More time points may be required for further investigation of the mechanism of action of PD. In addition, resveratrol was not included in the present study because it showed a therapeutic effect inferior to that of PD with regard to shock treatment according to our previous study [15]. Moreover, in this study, we found that PD increased Sirt1 protein levels; however, the transcriptional regulation of PD on Sirt1 was not tested, which will be examined in a future study.

## 5. Conclusion

The present study demonstrated that MD is present in RTECs after HS/R and is related to the SIRT1-p53 pathway. PD appears to be a new activator of SIRT1, which is probably one of the mechanisms involved in the mitochondrial-protection effect of RTECs against HS/R. Through SIRT1 activation, PD reduces H/R-induced apoptosis of HK-2 cells* via* upregulation of the SIRT1-p53 signaling pathway.

## Supplementary Material

Supplementary Figure 1. Molecular structure of polydatin (left) and resveratrol (right).

## Abbreviations

PD: Polydatin
MD: Mitochondrial dysfunction
SIRT1: Silent information regulator 1
RTECs: Renal tubular epithelial cells
H/R: Hypoxia/reoxygenation
HS/R: Hemorrhagic shock and reperfusion
AKI: Acute kidney injury
MAP: Mean arterial pressure
acetyl: Acetylated
HK: Human kidney
TEM: Transmission electron microscopy.

## References

[1] Nash K., Hafeez A., Hou S. (2002). Hospital-acquired renal insufficiency. *American Journal of Kidney Diseases*.

[2] Tran M., Tam D., Bardia A. (2011). PGC-1*α* promotes recovery after acute kidney injury during systemic inflammation in mice. *The Journal of Clinical Investigation*.

[3] Houtkooper R. H., Pirinen E., Auwerx J. (2012). Sirtuins as regulators of metabolism and healthspan. *Nature Reviews Molecular Cell Biology*.

[4] Vaziri H., Dessain S. K., Ng E. E. (2001). *hSIR2^SIRT1^* functions as an NAD-dependent p53 deacetylase. *Cell*.

[5] Luo J., Nikolaev A. Y., Imai S.-I. (2001). Negative control of p53 by Sir2*α* promotes cell survival under stress. *Cell*.

[6] Langley E., Pearson M., Faretta M. (2002). Human SIR2 deacetylates p53 and antagonizes PML/p53-induced cellular senescence. *The EMBO Journal*.

[7] Howitz K. T., Bitterman K. J., Cohen H. Y. (2003). Small molecule activators of sirtuins extend Saccharomyces cerevisiae lifespan. *Nature*.

[8] Giovannini L., Migliori M., Longoni B. M. (2001). Resveratrol, a polyphenol found in wine, reduces ischemia reperfusion injury in rat kidneys. *Journal of Cardiovascular Pharmacology*.

[9] Kim D. H., Jung Y. J., Lee J. E. (2011). SIRT1 activation by resveratrol ameliorates cisplatin-induced renal injury through deacetylation of p53. *The American Journal of Physiology—Renal Physiology*.

[10] Zhang C., Feng Y., Qu S. (2011). Resveratrol attenuates doxorubicin-induced cardiomyocyte apoptosis in mice through SIRT1-mediated deacetylation of p53. *Cardiovascular Research*.

[11] Sheng C., Yu Y.-H., Zhao K.-S., Qin W., Wang C.-H. (2011). Hypotensive resuscitation combined with polydatin improve microcirculation and survival in a rabbit model of uncontrolled hemorrhagic shock in pregnancy. *Journal of Surgical Research*.

[12] Miao Q., Wang S., Miao S., Wang J., Xie Y., Yang Q. (2011). Cardioprotective effect of polydatin against ischemia/reperfusion injury: roles of protein kinase C and mito K_ATP_ activation. *Phytomedicine*.

[13] Li X.-H., Gong X., Zhang L. (2013). Protective effects of polydatin on septic lung injury in mice via upregulation of HO-1. *Mediators of Inflammation*.

[14] Wang X., Song R., Bian H. N., Brunk U. T., Zhao M., Zhao K.-S. (2012). Polydatin, a natural polyphenol, protects arterial smooth muscle cells against mitochondrial dysfunction and lysosomal destabilization following hemorrhagic shock. *The American Journal of Physiology—Regulatory Integrative and Comparative Physiology*.

[15] Wang X., Song R., Chen Y., Zhao M., Zhao K.-S. (2013). Polydatin—a new mitochondria protector for acute severe hemorrhagic shock treatment. *Expert Opinion on Investigational Drugs*.

[16] Li P., Wang X., Zhao M., Song R., Zhao K. (2015). Polydatin protects hepatocytes against mitochondrial injury in acute severe hemorrhagic shock via SIRT1-SOD_2_ pathway. *Expert Opinion on Therapeutic Targets*.

[17] Zeng Z., Chen Z., Xu S., Song R., Yang H., Zhao K. (2015). Polydatin alleviates small intestine injury during hemorrhagic shock as a SIRT1 activator. *Oxidative Medicine and Cellular Longevity*.

[18] Yu L., Sun Y., Cheng L. (2014). Melatonin receptor-mediated protection against myocardial ischemia/reperfusion injury: role of SIRT1. *Journal of Pineal Research*.

[19] Nakanishi T., Fukushi A., Sato M. (2011). Functional characterization of apical transporters expressed in rat proximal tubular cells (PTCs) in primary culture. *Molecular Pharmaceutics*.

[20] Weiland C., Ahr H. J., Vohr H. W., Ellinger-Ziegelbauer H. (2007). Characterization of primary rat proximal tubular cells by gene expression analysis. *Toxicology in Vitro*.

[21] Mattila P. M., Nietosvaara Y. A., Ustinov J. K., Renkonen R. L., Hayry P. J. (1989). Antigen expression in different parenchymal cell types of rat kidney and heart. *Kidney International*.

[22] Gao Y., Zeng Z., Li T. (2015). Polydatin inhibits mitochondrial dysfunction in the renal tubular epithelial cells of a rat model of sepsis-induced acute kidney injury. *Anesthesia & Analgesia*.

[23] Beier U. H., Wang L., Han R., Akimova T., Liu Y., Hancock W. W. (2012). Histone deacetylases 6 and 9 and sirtuin-1 control Foxp^3+^ regulatory T cell function through shared and isoform-specific mechanisms. *Science Signaling*.

[24] Shimizu H., Bolati D., Adijiang A. (2010). Senescence and dysfunction of proximal tubular cells are associated with activated p53 expression by indoxyl sulfate. *American Journal of Physiology—Cell Physiology*.

[25] Kelly K. J., Plotkin Z., Vulgamott S. L., Dagher P. C. (2003). P53 mediates the apoptotic response to GTP depletion after renal ischemia-reperfusion: protective role of a p53 inhibitor. *Journal of the American Society of Nephrology*.

[26] McLaren B. K., Zhang P. L., Herrera G. A. (2004). P53 protein is a reliable marker in identification of renal tubular injury. *Applied Immunohistochemistry & Molecular Morphology*.

[27] Vaseva A. V., Moll U. M. (2009). The mitochondrial p53 pathway. *Biochimica et Biophysica Acta (BBA)—Bioenergetics*.

[28] Erster S., Mihara M., Kim R. H., Petrenko O., Moll U. M. (2004). In vivo mitochondrial p53 translocation triggers a rapid first wave of cell death in response to DNA damage that can precede p53 target gene activation. *Molecular and Cellular Biology*.

