# Supplementary material for: Polydatin Protecting Kidneys against Hemorrhagic Shock-Induced Mitochondrial Dysfunction via SIRT1 Activation and p53 Deacetylation
Source: Oxid Med Cell Longev. 2016 Feb 24;2016:1737185. doi: 10.1155/2016/1737185 (PMC4783550; doi:10.1155/2016/1737185)
Supplement: Supplementary file 1 — Supplementary Figure 1. Molecular structure of polydatin (left) and resveratrol (right). [file 1737185.f1.pdf]

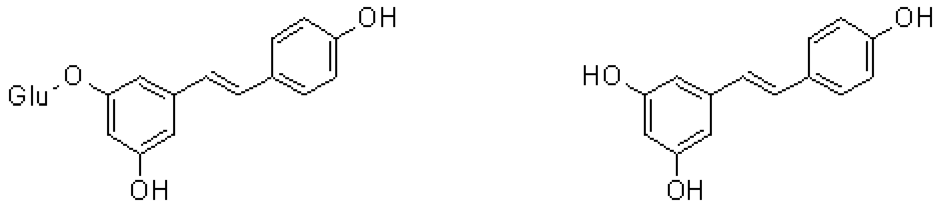

Supplementary Figure 1. Molecular structure of polydatin (left) and resveratrol (right)

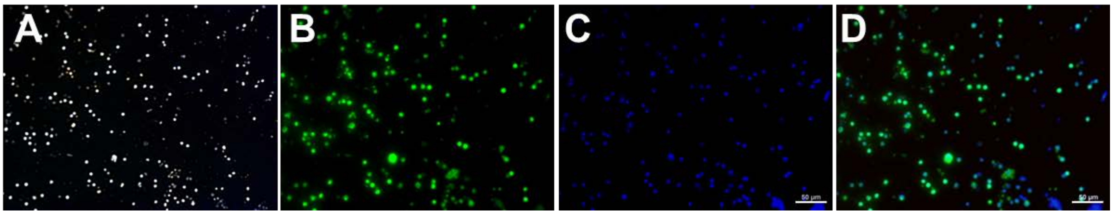

Supplementary Figure 2. Identification of renal tubular epithelial cells (RTECs). Original magnification, ×200. (A) Bright field of detected cells; (B) cells were stained with antibodies against cytokeratin-18 (CK-18, marked with fluorescein isothiocyanate (FITC)), which is a marker of RTECs; (C) cells were stained with the nuclear dye Hoechst 33342; (D) merged image from CK-18–FITC and Hoechst. These images demonstrated that isolated cells were RTECs of high purity (>90%).

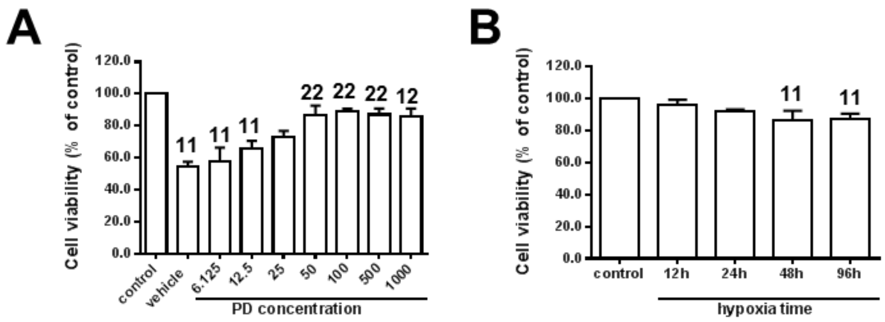

Supplementary Figure 3. Determination of viability of human kidney (HK)-2 cells through exposure to different polydatin (PD) doses and different durations of hypoxia/reoxygenation (H/R). (A) Effect of different doses of PD on viability of HK-2 cells. An increased concentration of PD resulted in superior cell-protective effects against H/R injury. When PD concentration >50 μM, cell viability tended to stabilize. When PD concentration >1000 μM, cell viability was suppressed partially. (B) Viability of the HK-2 line at different durations of hypoxia. $^{11}P<0.01$, $^{1}P<0.05$ compared with the value of the control group; $^{22}P<0.01$, $^{2}P<0.05$ compared with the value of the vehicle group.

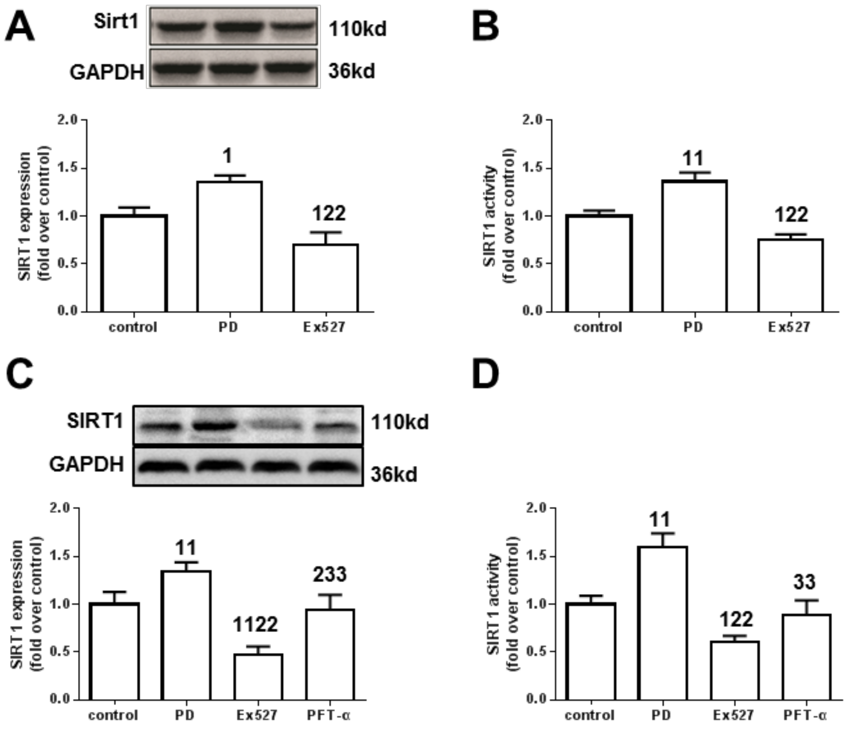

Supplementary Figure 4. Effect of administration of polydatin (PD) on the protein expression and activity of silent information regulator (SIRT)1 protein in the renal cortex tissue of healthy rats and normal human kidney (HK)-2 cells. (A) Protein expression and (B) activity of SIRT1 in the renal cortex of healthy rats. (C) Protein expression and (D) activity of SIRT1 in normal HK-2 cells. Ex527 was administered at 5 mg/kg body weight of rats and at 10 μM for HK-2 cells. PFT-α was administered at 10 μM for HK-2 cells. ^{11}P<0.01, ^{1}P<0.05 compared with the value of the control group; ^{22}P<0.01 compared with the value of the PD group; ^{33}P<0.01, ^{3}P<0.05 compared with the value of the PFT-α group. GAPDH, glyceraldehyde 3-phosphate dehydrogenase; PFT-α, pifithrin-α.
